# CSF α-synuclein seed amplification assay positivity identifies a distinct soluble amyloid-β profile linked to fibrillar amyloid burden and structural neurodegeneration

**DOI:** 10.21203/rs.3.rs-10731083/v1

**Published:** 2026-08-27

**Authors:** Norberto Rodriguez-Espinosa, Alzheimer’s Disease Neuroimaging Initiative

**Affiliations:** Universidad de La Laguna

**Keywords:** alpha-synuclein, seed amplification assay, amyloid-beta, Alzheimer’s disease, cerebrospinal fluid, neurodegeneration

## Abstract

**Purpose.:**

To determine whether cerebrospinal fluid (CSF) α-synuclein seed amplification assay (SAA) positivity identifies a reproducible soluble amyloid-β (Aβ) configuration and whether its dimensions carry distinct information about downstream pathology and neurodegeneration.

**Methods.:**

In ADNI, 72 participants (13 SAA-positive [SAA+]) had same-day BACE1 activity, sAPPβ, and mass-spectrometry Aβ38/Aβ40/Aβ42. T captured common Aβ38/Aβ40 variation and D42 Aβ42 depletion relative to Aβ38/Aβ40. A one-factor measurement-error model tested common-factor compatibility. The pattern was then tested in 560 participant-disjoint individuals (131 SAA+), with T and D42 reconstructed de novo, and related to amyloid PET, clinical change, whole-brain MRI, and tau PET.

**Results.:**

In the proximal cohort, SAA + was associated with lower T (β=−0.549 SD, 95% CI −1.005 to −0.093), but this effect was compatible with the one-factor model (directional compatibility probability = 0.288). In the participant-disjoint cohort, SAA + was associated with lower T (β=−0.267 SD) and higher D42 (β = 0.206 SD); their direct contrast was - 0.473 SD. Higher D42 was associated with subsequent amyloid burden, faster 0-2.5-year clinical worsening, greater whole-brain loss, and subsequent tau burden; lower T independently predicted faster whole-brain loss.

**Conclusions.:**

SAA positivity identifies a reproducible T-low/D42-high soluble-Aβ configuration. The preferential association of D42 with fibrillar amyloid, tau burden, and clinical worsening links the Aβ42-selective component to the AD pathological cascade, while the overall pattern points to altered post-β-cleavage APP/Aβ handling.

## 1 Introduction

Alpha-synuclein (αSyn) pathology frequently co-occurs with Alzheimer’s disease (AD) pathology, but its place within the molecular architecture of human AD remains incompletely resolved. CSF αSyn seed amplification assays (SAA) now permit in-vivo detection of seeding-competent misfolded αSyn with substantially greater pathological specificity than total αSyn concentration. In ADNI, αSyn-SAA positivity is present across the clinical AD spectrum and has been associated with biomarker burden and subsequent cognitive progression (Tosun et al. 2024a, b). Other cohorts have linked αSyn copathology to amyloid-associated tau accumulation and structural degeneration (Franzmeier et al. 2025; Esteller-Gauxax et al. 2026; Mak et al. 2026). These findings establish αSyn-SAA as a biologically meaningful copathology marker, but they do not show where αSyn-associated variation is expressed within APP processing and soluble Aβ measurements.

This question is potentially informative because the measurable APP/Aβ system spans observables with different biological proximity. CSF BACE1 activity and sAPPβ are proximal APP-processing readouts, whereas secreted Aβ38, Aβ40, and Aβ42 integrate additional unmeasured processing, trafficking, release, binding, and clearance steps. Earlier studies in Lewy body dementia reported reductions in shorter soluble Aβ species and examined sAPP isoforms, but did not combine molecular αSyn-SAA status with BACE1 activity, sAPPβ, and same-assay Aβ38/Aβ40/Aβ42 in a common analytical framework (Mulugeta et al. 2011; van Steenoven et al. 2019). Ratios such as Aβ42/Aβ40 and Aβ42/Aβ38 also indicate that Aβ42 can carry information relative to the background abundance of shorter soluble species rather than only as an absolute concentration (Janelidze et al. 2016).

We therefore decomposed soluble Aβ variation into two prespecified analytical dimensions: T, representing common Aβ38/Aβ40 variation, and D42, representing Aβ42 depletion relative to the joint Aβ38/Aβ40 state. The analysis followed an explicit evidential hierarchy. First, we tested αSyn-SAA associations in participants with same-day BACE1, sAPPβ, and Aβ38/Aβ40/Aβ42. Second, we challenged the strongest proximal-to-soluble interpretation with a fitted one-factor measurement-error model designed to reproduce the overall SAA-T displacement without any biomarker-specific SAA effect. Third, we tested the soluble pattern in 560 participants completely disjoint from the cohort in which the analytical coordinates had originally been developed. Finally, we examined how the SAA-associated coordinates related to later fibrillar amyloid, clinical progression, whole-brain atrophy, and tau PET. The objective was not to assign causal responsibility to a biomarker, but to determine whether molecular αSyn positivity marks a reproducible reconfiguration of soluble Aβ information and whether the implicated dimensions have distinct external correlates.

## 2 Methods

### 2.1 Study design, ADNI data source, and ethics

This was a secondary observational analysis of data from the Alzheimer’s Disease Neuroimaging Initiative (ADNI), a longitudinal multicentre study (Weiner et al. 2010). Data used in the preparation of this article were obtained from the ADNI database (adni.loni.usc.edu). ADNI was launched in 2003 as a public-private partnership led by Principal Investigator Michael W. Weiner, MD, to evaluate whether serial magnetic resonance imaging, positron emission tomography, other biological markers, and clinical and neuropsychological assessment can be combined to measure progression across mild cognitive impairment and Alzheimer’s disease. Each ADNI phase is implemented under Institutional Review Board-approved protocols at participating sites, and participants or authorized representatives provide informed consent. The present study was a secondary analysis of ADNI data released to approved investigators under the ADNI Data Use Agreement; no additional local ethics committee review was obtained for this secondary analysis. The analysis involved no new participant contact or biospecimen collection. The contributing ADNI phases are registered at ClinicalTrials.gov (NCT00106899, NCT01078636, NCT01231971, and NCT02854033).

Analyses used ADNI releases available through 12 August 2026. Participant enrichment was performed by left joins from explicit backbones; missing measurements produced analysis-specific eligibility masks rather than silent deletion from the parent cohort. No missing biomarker or outcome values were imputed. Clinical CN/MCI/AD labels were treated as syndromic descriptors rather than etiologic diagnoses.

### 2.2 Analytical chronology and evidential hierarchy

**Figure 1:**
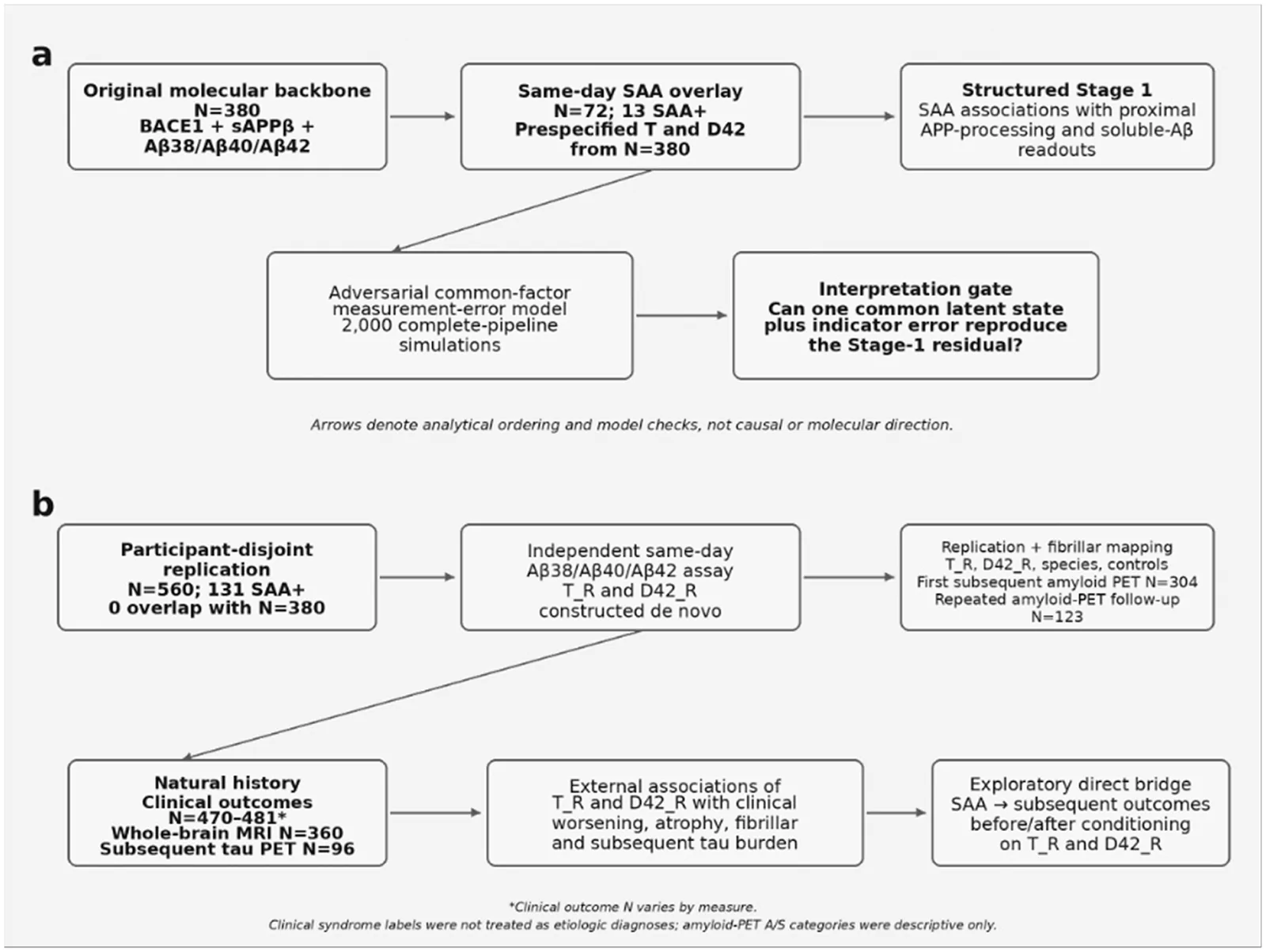
Analytical hierarchy and inference boundaries. (a) The same-day proximal cohort overlays αSyn-SAA status on a prespecified N=380 molecular backbone containing BACE1 activity, sAPPβ, and same-assay Aβ38/Aβ40/Aβ42. The Stage-1 residual association is challenged with an adversarial common-factor measurement-error model. (b) A participant-disjoint exact same-day replication reconstructs T_R and D42_R de novo, followed by fibrillar mapping, natural-history analyses, and an explicitly exploratory direct SAA-to-outcome bridge. Arrows denote analytical ordering and model checks, not causal or molecular direction

An initial exploratory same-day αSyn-SAA signal was observed before the formal Stage-1 specification. That signal motivated a locked analysis plan for the N = 72 same-day proximal cohort; Stage 1 is therefore structured and reproducible but is not presented as independent confirmatory evidence. A participant-disjoint replication cohort was subsequently defined, fully excluded from the original N = 380 molecular backbone, and prespecified before inspection of its SAA-soluble associations. The common-factor challenge, participant-disjoint replication, fibrillar mapping, and natural-history analyses were likewise executed under dated specifications. Direct SAA-to-outcome models were added later as an explicitly exploratory bridge after the separate SAA-to-coordinate and coordinate-to-outcome patterns were already known. Figure 1 summarizes this hierarchy.

### 2.3 αSyn-SAA classification

CSF αSyn-SAA results were obtained from the ADNI Amprion SAA release described by Tosun et al. (2024a, b). Results coded as Detected-1 or Detected-2 were classified SAA positive (S+); Not_Detected was classified SAA negative (S−). Indeterminate results were retained in participant-level masters with missing S status and were never recoded as negative. The primary proximal analysis required SAA and the molecular landmark to occur on the same day. The participant-disjoint replication likewise required exact same-day SAA and Aβ38/Aβ40/Aβ42 measurements.

### 2.4 Proximal APP-processing and soluble Aβ measurements

CSF BACE1 activity and sAPPβ were obtained from the Biomarkers Consortium ADNI CSF Targeted Proteomics Project. In the source project, BACE1 activity and sAPPβ were measured concurrently from aliquots obtained from the same vial at the same thaw. BACE1 activity was quantified using a two-step enzyme/substrate assay followed by immunodetection, and sAPPβ using a specific immunoassay; recombinant standards were used for quantification and both measures were reported in pM (Wu et al. 2008, 2011). These variables are treated here as proximal APP-processing readouts, not as direct measurements of intracellular APP flux.

The original N = 380 molecular backbone used University of Pennsylvania 2D-UPLC tandem mass-spectrometry Aβ38, Aβ40, and Aβ42. All three peptides were quantified within the same assay; reported concentrations represented the average of duplicate analyses of 0.1-mL CSF aliquots and were expressed in pg/mL (Lame et al. 2011; Korecka et al. 2014). BACE1/sAPPβ and MS Aβ measurements were matched by CSF collection date but are not described as originating from one common aliquot. The participant-disjoint replication used the independent UPENNMSMSABETA2 release, which measured Aβ38/Aβ40/Aβ42 by LC-MS/MS in ADNI GO/2 samples (Korecka et al. 2020). All concentration variables were log-transformed before standardization.

### 2.5 Soluble Aβ coordinate definitions

The original analytical coordinates were constructed without PET, SAA, BACE1, or sAPPβ. T represents the common standardized variation of Aβ38 and Aβ40:

T=z{[z(logAβ38)+z(logAβ40)]∕2}

T is an analytical common shorter-Aβ concentration coordinate and is not interpreted as a molecular production or flux rate. The symbol T refers only to this Aβ38/Aβ40 coordinate and is unrelated to the T (tau) category of AT(N).

D42 represents lower Aβ42 than expected from the joint Aβ38/Aβ40 state. Expected log Aβ42 was estimated using 50 repeated participant-level five-fold out-of-fold splits; each participant’s predictions were averaged across out-of-fold appearances, the residual was sign-reversed, and the result standardized:

$$D42\;=-z\{log\;A\beta \;42\;-\hat{E}OOF[log\;A\beta \;42\;|\;log\;A\beta \;38\;,\;log\;A\beta \;40\;]\}$$

Positive D42 therefore denotes greater Aβ42-relative depletion. Out-of-fold construction reduces self-prediction of the generated coordinate (Stone 1974). A secondary Aβ38:Aβ40 balance coordinate was G38:40 = z{[z(log Aβ38) - z(log Aβ40)]/2}. D38, used as a negative-control residual in the replication cohort, was defined analogously as −z{log Aβ38 - ÊOOF[log Aβ38 ∣ log Aβ40]}. These coordinates are analytical decompositions of measured soluble species and are not direct measures of aggregation, trafficking, γ-secretase activity, sequestration, or clearance.

In Stage 1, T, D42, BACE1, sAPPβ, age, and education were retained exactly as prespecified from the full N = 380 backbone and were not restandardized within the SAA subset. In the participant-disjoint N = 560 cohort, T_R, D42_R, G38:40_R, and D38_R were constructed de novo within that cohort using the same mathematical definitions and a new 50×5 out-of-fold sequence. Accordingly, replication refers to participant-independent replication of the analytical structure rather than application of coefficients estimated in N = 380.

### 2.6 Same-day proximal cohort and Stage-1 models

The original molecular backbone contained 380 unique participants with contemporaneous BACE1 activity, sAPPβ, and MS Aβ38/Aβ40/Aβ42 plus age, sex, APOE ε4 dosage, education, and clinical syndrome label. Exact same-day matching to interpretable αSyn-SAA yielded N = 72 participants (13 SAA+). The primary model was ordinary least squares (OLS) with HC3 heteroskedasticity-robust covariance (MacKinnon and White 1985): T ~ SAA status + BACE1 + sAPPβ + age + sex + APOE ε4 dosage + education + MCI + AD syndrome indicators. Secondary models placed SAA status against BACE1, sAPPβ conditional on BACE1, D42, G38:40, and the three individual soluble Aβ species using the frozen covariate structure. A prespecified paired contrast compared the SAA coefficients for T and D42. Supportive robustness included 2,000 participant bootstraps and leave-one-SAA-positive-out refitting. A diagnosis-free sensitivity removed MCI/AD indicators; a separate timing sensitivity included interpretable SAA obtained 0-365 days after the molecular landmark, with SAA lag included as a covariate. Because those later SAA measurements cannot precede the molecular state, this timing sensitivity was interpreted only as baseline biomarker differences according to SAA status detected within one year.

### 2.7 Adversarial common-factor measurement-error challenge

We explicitly tested whether the Stage-1 residual SAA-T association could arise without a special perturbation between the proximal readouts and secreted Aβ. In the full N = 380 backbone, log BACE1, log sAPPβ, log Aβ38, log Aβ40, and log Aβ42 were standardized, regressed on the frozen covariates, residualized, rescaled to unit residual variance, and fitted with a one-component linear-Gaussian factor model. Fixed Stage-1 SAA labels were permitted to shift only the single latent factor; direct SAA effects on individual biomarkers were prohibited. The latent SAA shift was calibrated by deterministic bisection so that the mean simulated reduced SAA-T association, before BACE1/sAPPβ adjustment, matched the observed reduced association within 0.01 SD.

Each of 2,000 final simulations regenerated all five biomarkers, restored their covariate-dependent means and observed residual scales, reconstructed T and the full 50×5 out-of-fold D42 pipeline, and refitted the frozen Stage-1 models. The primary compatibility statistic was the adjusted SAA-T coefficient. A directional empirical probability was calculated with the + 1 correction (Phipson and Smyth 2010). This model was intentionally favorable to the one-factor explanation and is interpreted only as a specific measurement-geometry benchmark, not as a comprehensive biological null.

### 2.8 Participant-disjoint replication and assay robustness

The primary replication required exact same-day interpretable SAA and UPENNMSMSABETA2 Aβ38/Aβ40/Aβ42, positive nonmissing peptide concentrations, and exclusion of every participant present anywhere in the original N = 380 backbone. This yielded N = 560 participants, including 131 SAA+, with zero participant overlap with N = 380. Primary covariates were age, sex, APOE ε4 dosage, and education. Clinical diagnosis was deliberately excluded from the primary replication because it is syndromic and may itself be influenced by copathology; a nearest-diagnosis sensitivity was prespecified.

The primary replication model was T_R ~ SAA status + age + sex + APOE ε4 dosage + education with HC3 covariance. The key secondary localization test was the direct coefficient contrast β(SAA→T_R) - β(SAA→D42_R), estimated as the SAA coefficient in a model with T_R-D42_R as the outcome and the identical covariate matrix. This avoids inferring different effects merely because one P value is significant and another is not. Secondary models evaluated D42_R, G38:40_R, standardized log Aβ38/Aβ40/Aβ42, and D38_R. A 500-replicate complete-pipeline participant bootstrap reconstructed scaling, T_R, out-of-fold D42_R, the primary model, and the direct contrast. A separate Meso Scale same-day sample (N = 82; 16 SAA+) tested assay robustness but was not considered an independent replication because 77 participants overlapped the primary N = 560 replication.

### 2.9 Amyloid PET and descriptive A/S biological phenotyping

Amyloid PET was obtained from the UC Berkeley 6-mm summary release and expressed on the Centiloid scale (Klunk et al. 2015). Analyses required a passing release quality-control flag, valid scan date, and nonmissing Centiloid value; duplicate participant/date/tracer records were resolved by the latest processing record. In the N = 560 backbone, the primary fibrillar model used the first quality-controlled amyloid PET strictly after the SAA/MS landmark and included SAA status, T_R, D42_R, time to PET, age, sex, APOE ε4 dosage, and education. A prespecified 2-df Wald test jointly assessed SAA×T_R and SAA×D42_R interactions. For repeated amyloid PET, the first subsequent scan became a landmark covariate and was excluded from outcomes; later scans were analyzed using Gaussian generalized estimating equations (GEE) with robust covariance, with a prespecified inverse-probability-weighted sensitivity for repeated-PET availability (Liang and Zeger 1986).

Separately, and only for descriptive biological phenotyping, SAA S status was cross-classified with the nearest eligible amyloid-PET A status within ± 365 days according to the 2024 Alzheimer’s Association biological taxonomy (Jack et al. 2024). A + was defined by the UC Berkeley tracer-specific AMYLOID_STATUS variable, A- by AMYLOID_STATUS = 0, and A? when no eligible contemporaneous PET was available. This A/S layer was never used to select, restrict, or adjust the primary soluble analyses and did not redefine clinical syndrome labels.

### 2.10 Natural-history outcomes

Natural-history analyses were prespecified before outcome associations were inspected. The full N = 380 architecture cohort retained standardized BACE1, sAPPβ, T, and D42 as joint predictors; the participant-disjoint N = 560 cohort provided replication for T_R and D42_R.

Clinical outcomes were CDR Sum of Boxes (CDR-SB; primary), ADAS-Cog13, Functional Activities Questionnaire (FAQ), and MMSE sign-reversed so that higher values consistently represented worse function. Baseline was the closest valid assessment within ± 180 days of the biomarker landmark; observations strictly after baseline through 5 years were retained. Follow-up was prespecified with two piecewise temporal intervals: 0-2.5 years and 2.5-5 years after the clinical baseline. These intervals are modeling periods only and are not diagnostic categories or biological disease-stage labels. Gaussian GEE with exchangeable working correlation and robust covariance included the baseline outcome, biomarker-to-clinical-baseline gap, demographic covariates, time terms, all available analytical components, and component×time interactions. Primary estimands were additional outcome worsening per year per 1-SD component within each follow-up interval.

Structural neurodegeneration was assessed with Fox Lab KN-BSI whole-brain longitudinal MRI (Leung et al. 2010). Quality-controlled BSI pairs were anchored to the base scan closest to the biomarker landmark within ± 365 days. Cumulative percentage whole-brain loss was calculated as 100×KN-BSI/base brain volume. Because change is zero at the pair baseline by construction, the GEE rate model was constrained through zero and used the same 0-2.5-year and 2.5-5-year temporal intervals.

Tau PET used the UC Berkeley non-partial-volume-corrected meta-temporal SUVR as the primary measure; partial-volume-corrected values were a sensitivity. The first quality-controlled tau scan strictly after the biomarker landmark was modeled using OLS-HC3 with time-to-tau and demographic covariates. N = 380 tau analysis was explicitly exploratory because of small availability. In N = 560, T_R and D42_R were modeled jointly. For participants with repeated post-landmark tau PET, the first tau scan was treated as a landmark and excluded from subsequent outcomes; subsequent tau was analyzed with GEE including baseline tau and T_R×time and D42_R×time interactions. Prespecified restricted cubic-spline models with knots at the 10th, 50th, and 90th percentiles tested nonlinearity secondarily; no post-result shape selection was allowed.

### 2.11 Exploratory direct SAA-to-outcome bridge

After the separate Stage-2 SAA-to-T/D42 and Stage-3 T/D42-to-outcome results were known, we prespecified an additional exploratory bridge before inspecting direct SAA-to-outcome coefficients. For each 0-2.5-year clinical outcome, whole-brain MRI loss, and subsequent tau burden, Model A estimated the adjusted association of SAA status with the subsequent outcome. Model B added the already defined T_R and D42_R coordinates. Change in the SAA coefficient after T_R/D42_R conditioning is reported descriptively as shared prognostic information and is not interpreted as causal mediation.

### 2.12 Statistical reporting and multiplicity

All tests were two-sided. Cross-sectional OLS models used HC3 robust covariance. Longitudinal models used GEE robust sandwich covariance. No global multiplicity correction was applied across secondary and exploratory outcomes; inferential weight was placed on the prespecified primary hypotheses, direct contrasts, effect sizes with 95% confidence intervals, consistency across participant-disjoint analyses, and explicit model challenges. CDR-SB was the primary clinical outcome; ADAS-Cog13, FAQ, and MMSE were convergent secondary outcomes; whole-brain KN-BSI was the primary structural outcome; N = 380 tau analyses and direct SAA-to-outcome bridge models were explicitly exploratory. Complete numerical results for both prespecified longitudinal follow-up intervals (0-2.5 and 2.5-5 years), formal contrasts between those intervals, nonlinear tests, assay robustness, amyloid-PET weighting, tau-PET partial-volume correction, and exploratory bridge analyses are reported in Online Resource 1. Analyses were conducted in Python using numpy, pandas, statsmodels, scikit-learn, and openpyxl; figures were generated in Python using matplotlib. Scripts, seeds, preflight checks, and output hashes were retained for reproducibility.

### 2.13 Use of generative AI tools

ChatGPT (OpenAI) assisted within the analysis environment with code development and checking, statistical documentation and reproducibility review, language editing, and manuscript organization/formatting. The author controlled ADNI access and file transfer; the AI system had no independent access to restricted ADNI credentials. The author reviewed and approved all analytical decisions, code, outputs, interpretations, and manuscript text and takes responsibility for the work. The AI system is not an author.

## 3 Results

### 3.1 Cohorts and participant characteristics

The same-day proximal cohort included 72 participants, 13 (18.1%) SAA+, whereas the participant-disjoint replication included 560 participants, 131 (23.4%) SAA+, with zero overlap with the original N = 380 molecular backbone (Table 1; Fig. 1). The replication cohort was older in SAA+ than SAA− participants on average and contained a greater proportion of clinically diagnosed AD dementia, but clinical syndrome was not included in primary replication models. Exact same-day design eliminated ambiguity about whether SAA was measured before or after the soluble-species landmark in these two primary molecular analyses.

### 3.2 Same-day proximal analysis: SAA positivity is associated with lower common shorter-species variation

**Figure 2:**
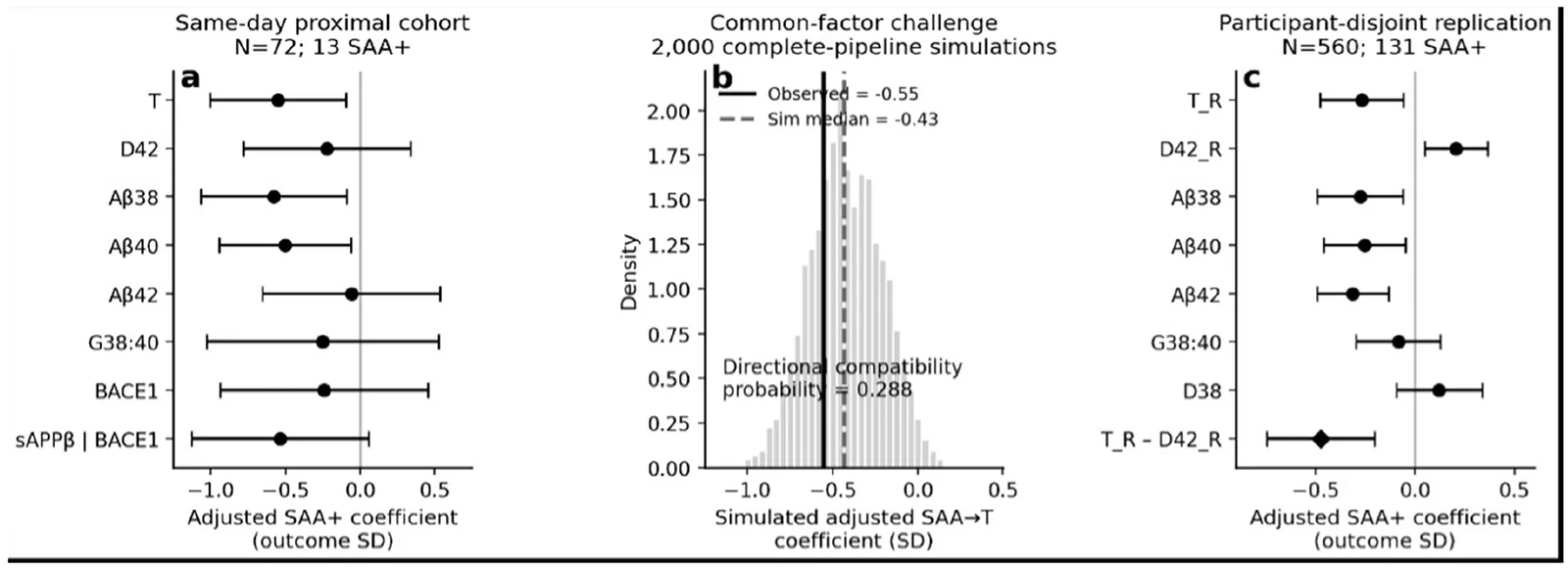
αSyn-SAA associations with soluble-Aβ architecture and the common-factor challenge. (a) Adjusted SAA+ coefficients and HC3 95% confidence intervals in the same-day proximal cohort (N=72; 13 SAA+). (b) Distribution of the adjusted SAA→T coefficient across 2,000 complete-pipeline simulations from the calibrated one-factor model; the solid vertical line is the observed Stage-1 coefficient and the dashed line is the simulated median. The directional simulation compatibility probability was 0.288. (c) Participant-disjoint replication (N=560; 131 SAA+) using de novo T_R and D42_R coordinates; the diamond denotes the direct T_R-D42_R coefficient contrast

In the N = 72 same-day proximal cohort, SAA + was associated with lower T after conditioning on BACE1, sAPPβ, age, sex, APOE ε4 dosage, education, and syndrome label (β=−0.549 SD, 95% CI −1.005 to −0.093, P = 0.0183; Fig. 2a; Table 2). The 2,000-replicate participant bootstrap was nearly identical (median −0.547, percentile interval −1.003 to −0.092), and leave-one-SAA-positive-out estimates remained negative throughout (−0.618 to −0.417). Removing diagnosis strengthened rather than weakened the estimate (β=−0.531, P = 0.0109). In the supportive 0-365-day timing sensitivity (N = 155; 34 SAA+), the adjusted T estimate remained negative (β=−0.352, 95% CI −0.673 to −0.032, P = 0.0312).

The secondary species results located the same-day signal predominantly in the shorter species: SAA + was associated with lower Aβ38 (−0.580 SD, P = 0.0198) and Aβ40 (−0.504 SD, P = 0.0255), whereas the Aβ42 estimate was near zero (−0.057 SD, P = 0.851). No clear D42 or G38:40 association was detected in this small cohort. Importantly, the prespecified T-versus-D42 coefficient contrast remained imprecise (bootstrap median - 0.350, 95% interval - 1.159 to 0.517); the same-day data therefore did not establish a selective T rather than D42 effect. SAA associations with BACE1 and sAPPβ were also imprecise (Table 2). Complete Stage-1 estimates and robustness analyses are provided in Online Resource 1.

### 3.3 A common-factor measurement-error model can reproduce the Stage-1 residual

The adversarial one-factor benchmark was deliberately calibrated to reproduce the observed reduced SAA-T association before BACE1/sAPPβ adjustment (observed - 0.890 SD; simulated mean - 0.886 SD). Under 2,000 complete-pipeline simulations, the adjusted SAA-T coefficient had median - 0.426 SD and a 2.5th-97.5th percentile range of −0.853 to 0.024 (Fig. 2b). The directional compatibility probability for an effect at least as negative as the observed - 0.549 SD was 0.288. Thus, conditioning on noisy BACE1/sAPPβ indicators can leave a Stage-1-sized SAA-T residual under a single common latent biomarker state. The residual association is therefore not, by itself, evidence that αSyn pathology perturbs a specific unmeasured intracellular interval.

### 3.4 Participant-disjoint replication reveals lower T and greater Aβ42-relative depletion

In N = 560 completely participant-disjoint individuals, the primary SAA-T_R association replicated: SAA + was associated with lower T_R (β=−0.267 SD, 95% CI −0.477 to −0.057, P = 0.0128; Fig. 2c). A 500-replicate complete-pipeline bootstrap gave median - 0.253 (percentile interval - 0.470 to −0.050), and adding the nearest clinical syndrome label as a sensitivity yielded β=−0.250 (P = 0.0215).

The replication also revealed a feature that was not resolved in N = 72: SAA + was associated with higher D42_R (β = 0.206 SD, 95% CI 0.048 to 0.365, P = 0.0108). The direct T_R-D42_R coefficient contrast was - 0.473 SD (95% CI −0.743 to −0.204, P = 0.00058), with a complete-pipeline bootstrap median of −0.457 (interval - 0.724 to −0.221). Species-level models showed lower Aβ38 (−0.276 SD), Aβ40 (−0.252 SD), and Aβ42 (−0.313 SD) in SAA+, with no clear G38:40_R shift and no clear D38_R negative-control shift (Table 2). The replicated configuration is therefore not a T-only phenotype: it combines lower common Aβ38/Aβ40 variation with additional Aβ42-relative depletion.

The independent Meso Scale assay robustness sample showed concordant directions but wide intervals: T=−0.281 SD (95% CI −0.867 to 0.305, P = 0.348), D42 = + 0.141 SD (95% CI −0.481 to 0.763, P = 0.657), and the direct T-D42 contrast=−0.422 SD (95% CI −1.194 to 0.350, P = 0.284; Online Resource 1). Because 77 of 82 participants overlapped the UPENN replication, these results support assay robustness only and were not counted as a second independent replication.

### 3.5 Descriptive biological A/S context

Within N = 560, nearest amyloid PET within ± 365 days was available for 470 participants. Descriptive A/S categories were A + S+ 81, A-S + 31, A?S + 19, A + S− 199, A-S− 159, and A?S− 71. These categories show that SAA positivity was not synonymous with amyloid-PET positivity or with a clinical AD syndrome. Because PET availability and A status could be consequences or correlates of the same Aβ system being studied, A/S categories were never used to restrict or adjust the soluble analyses. The complete descriptive A/S cross-classification is provided in Online Resource 1.

### 3.6 Subsequent fibrillar amyloid burden maps predominantly to D42 rather than SAA status

**Figure 3:**
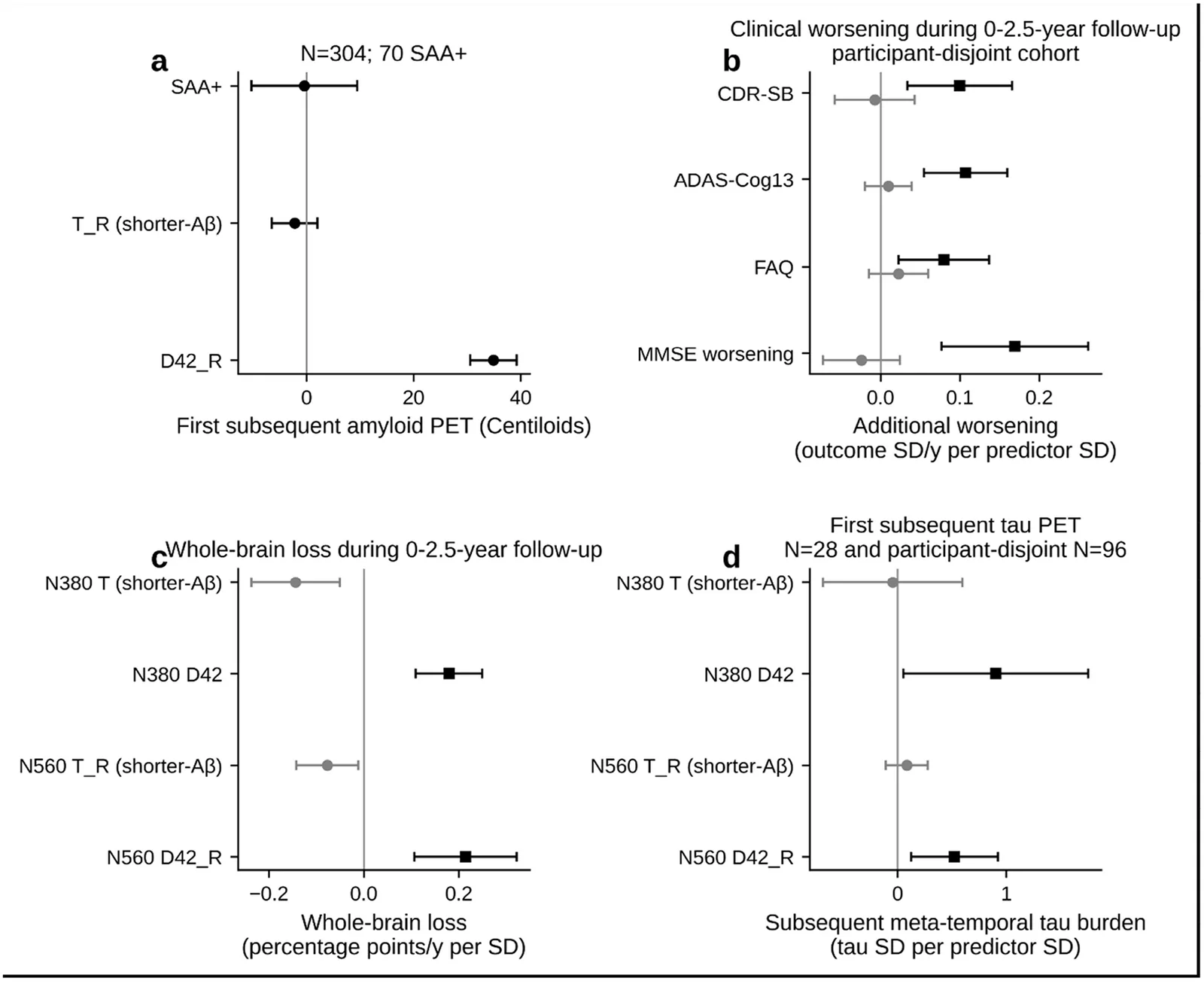
External associations of T and D42. (a) Joint associations of SAA status, T_R, and D42_R with first subsequent amyloid-PET Centiloids in N=304. (b) Clinical worsening during the 0-2.5-year follow-up interval in the participant-disjoint cohort, standardized by each outcome baseline SD; circles denote T_R and squares D42_R. (c) Whole-brain KN-BSI loss during the 0-2.5-year follow-up interval in N=380 and participant-disjoint N=560. (d) Joint associations with first subsequent meta-temporal tau-PET burden in N=380 and N=560. T denotes the common shorter-Aβ coordinate and is unrelated to AT(N) tau status

First quality-controlled amyloid PET after the same-day SAA/MS landmark was available in 304 replication participants, including 70 SAA+, at a median interval of 0.374 years. In the joint model, D42_R was strongly associated with subsequent fibrillar burden (+ 34.90 Centiloids per SD, 95% CI 30.57 to 39.23, P = 2.9×10^-56), whereas T_R (−2.19 Centiloids/SD, P = 0.314) and SAA status (−0.41 Centiloids, P = 0.935) carried little independent information (Fig. 3a; Table 3). The prespecified global SAA×T_R plus SAA×D42_R interaction test was null (P = 0.817), providing no evidence that SAA status altered the soluble-coordinate-to-PET mapping in this model. Complete model and interaction estimates are provided in Online Resource 1.

Among 123 participants with a first post-SAA amyloid-PET landmark and later PET follow-up, SAA×time was not detectable (−1.15 Centiloids/year, 95% CI −5.66 to 3.35, P = 0.617). Lower T_R predicted faster subsequent PET increase (T_R×time=−1.70 Centiloids/year per SD, 95% CI −2.50 to −0.90, P = 3.0×10^-5), while higher D42_R showed a positive, less robust slope association (+ 2.22 Centiloids/year per SD, 95% CI 0.02 to 4.41, P = 0.0476). Inverse-probability weighting preserved the T_R result (−1.66, 95% CI −2.53 to −0.79, P = 0.00019) and yielded a similar but slightly less precise D42_R estimate (+ 2.19, 95% CI −0.08 to 4.46, P = 0.0585; Online Resource 1).

### 3.7 Natural history: D42 carries the most consistent clinical and tau information, while T and D42 both inform structural loss

In the original N = 380 full-architecture cohort, higher D42 predicted faster worsening during the 0-2.5-year follow-up interval in CDR-SB (+ 0.226 points/year per SD, P = 0.0017), ADAS-Cog13 (+ 0.452/year, P = 0.038), and FAQ (+ 1.058/year, P = 2.5×10^-7), with a directionally similar MMSE-worsening estimate (+ 0.284/year, P = 0.056). T did not show a coherent conditional clinical-progression signal during this interval, and BACE1/sAPPβ showed little reproducible incremental prognostic information after the soluble coordinates were included.

The participant-disjoint N = 560 cohort reproduced the D42_R clinical pattern during the 0-2.5-year follow-up interval across all four outcomes: CDR-SB + 0.241/year (P = 0.0030), ADAS-Cog13 + 1.215/year (P = 6.1×10^-5), FAQ + 0.604/year (P = 0.0061), and MMSE worsening + 0.568/year (P = 0.00033) per SD D42_R (Fig. 3b; Table 3). T_R remained near null for clinical progression. Formal D42_R-versus-T_R contrasts supported stronger D42_R associations during this interval for CDR-SB, ADAS-Cog13, and MMSE; FAQ was directionally similar but imprecise.

Whole-brain structural loss showed a different two-dimensional pattern. In N = 380, lower T and higher D42 independently predicted faster KN-BSI whole-brain loss during the 0-2.5-year follow-up interval (T=−0.144 and D42 = + 0.180 percentage points/year per SD; P = 0.0025 and 5.2×10^-7, respectively). The same directions replicated in N = 560 (T_R=−0.077, P = 0.021; D42_R = + 0.214, P = 9.3×10^-5; Fig. 3c). Thus the SAA-associated T-low/D42-high region aligns with the two-dimensional direction of faster structural loss, although this cross-analysis alignment is not evidence of mediation.

Subsequent tau burden again concentrated more strongly in D42. In the exploratory N = 380 subset with subsequent tau PET (N = 28), D42 was associated with higher meta-temporal tau burden (+ 0.904 tau SD per SD, 95% CI 0.054 to 1.754, P = 0.037), whereas T was - 0.043 (95% CI −0.685 to 0.599, P = 0.895). In the participant-disjoint N = 560 tau subset (N = 96; median landmark-to-tau interval 4.31 years), D42_R was associated with + 0.525 tau SD (95% CI 0.125 to 0.924, P = 0.010) while T_R was + 0.086 (95% CI −0.107 to 0.279, P = 0.380); the direct contrast favored D42_R (P = 0.0493). The partial-volume-corrected sensitivity showed the same ordering (N = 89: T_R = + 0.056, 95% CI −0.150 to 0.262, P = 0.592; D42_R = + 0.574, 95% CI 0.165 to 0.983, P = 0.0059; T_R-D42_R contrast=−0.518, 95% CI −0.976 to −0.060, P = 0.0268; Fig. 3d; Online Resource 1). Among N = 47 with repeated post-landmark tau PET, neither T_R nor D42_R clearly predicted subsequent tau accumulation rate.

Prespecified estimates for the 2.5-5-year follow-up interval were fully retained and are reported in Online Resource 1. In N = 560, D42_R clinical estimates in this interval were smaller and imprecise (CDR-SB + 0.126/year, P = 0.265; ADAS-Cog13 + 0.102/year, P = 0.778; FAQ + 0.294/year, P = 0.260; MMSE worsening + 0.259/year, P = 0.172), while T_R estimates were also imprecise. N = 380 contained several isolated nominal associations during 2.5-5 years, including D42 with ADAS-Cog13 and T with FAQ and whole-brain loss, but these patterns were not reproduced in N = 560. Some formal contrasts between the 0-2.5-year and 2.5-5-year MRI coefficients were nominally significant, yet their directions were not coherent across the two cohorts. Prespecified nonlinear models likewise did not reveal a stable exposure-response shape that replicated across cohorts or outcome variants. Overall, the complete 5-year follow-up did not establish a reproducible change in which analytical component carried the strongest prognostic information across the two temporal intervals.

### 3.8 Exploratory direct SAA-to-outcome bridge: clearest association with structural loss

Direct SAA-to-outcome models in the N = 560 backbone were prespecified only after the preceding SAA-coordinate and coordinate-outcome results were known and are therefore explicitly exploratory. During the 0-2.5-year follow-up interval, SAA + was not clearly associated with CDR-SB, ADAS-Cog13, FAQ, or MMSE progression; confidence intervals were wide and included zero (Table 4). Similarly, SAA status was not detectably associated with first subsequent meta-temporal tau burden or with post-landmark tau accumulation rate. Complete estimates for both longitudinal follow-up intervals, tau-PVC, and tau-progression bridge analyses are provided in Online Resource 1.

The clearest direct association was structural. Among 360 participants with longitudinal whole-brain BSI, including 85 SAA+, SAA + was associated with + 0.233 percentage points/year faster whole-brain loss during the 0-2.5-year follow-up interval (95% CI 0.041 to 0.424, P = 0.0171). Adding T_R and D42_R reduced the SAA coefficient to + 0.140 (95% CI −0.047 to 0.327, P = 0.142), a descriptive attenuation of approximately 40%. This attenuation is compatible with shared prognostic information between the SAA-associated soluble configuration and structural loss but is not a mediation estimate.

## 4 Discussion

Viewed as a whole, the findings suggest that αSyn-SAA positivity is not simply superimposed on Alzheimer biology as an independent categorical copathology, but is accompanied by a reproducible reorganization of soluble Aβ measurements. In participant-independent data, SAA+ individuals occupied a T-low/D42-high region: common Aβ38/Aβ40 variation was lower, while Aβ42 showed additional depletion relative to that shorter-peptide background. This is not equivalent to a uniform downward shift of all soluble Aβ species. Instead, αSyn positivity marks a change in the internal organization of the soluble Aβ system, whose dimensions show different relationships with fibrillar amyloid, tau burden, clinical progression, and structural neurodegeneration.

This pattern extends observations from Lewy body disorders into a molecularly defined αSyn-positive state. Dementia with Lewy bodies has been associated with reductions in CSF Aβ38, Aβ40, and Aβ42, including shorter-species reductions not fully explained by concomitant AD pathology (van Steenoven et al. 2019), while relationships between sAPP isoforms and soluble Aβ species differ between Lewy body and AD groups (Mulugeta et al. 2011). The present results refine those observations using αSyn-SAA rather than a clinical Lewy body diagnosis: SAA positivity combined lower common shorter-Aβ variation with an additional Aβ42-relative component. The absence of a clear G38:40 shift further argues against simple redistribution between Aβ38 and Aβ40.

The most informative inference concerns where within APP/Aβ biology this reorganization may be expressed. BACE1 activity and sAPPβ lie relatively close to proximal β-cleavage, whereas secreted Aβ38, Aβ40, and Aβ42 integrate subsequent processing, trafficking, release, binding, aggregation, and clearance. The Stage-1 residual alone cannot localize the responsible process because the fitted common-factor model can reproduce a residual association of similar magnitude; thus localization cannot rest on the SAA–T residual after BACE1/sAPPβ adjustment. The participant-disjoint T-low/D42-high configuration imposes a stronger constraint: any explanation must generate lower common Aβ38/Aβ40 variation together with additional Aβ42-relative depletion without requiring a comparably large proximal shift. This makes a simple uniform change in β-secretase activity incomplete and points most plausibly downstream of proximal β-cleavage, where β-cleaved APP products are trafficked and processed and Aβ species are sorted and disposed. Candidate mechanisms include endosomal–secretory APP-CTF trafficking, access to and processing by γ-secretase, vesicular export, intracellular degradation, and species-selective retention or extracellular disposition. Experimental αSyn overexpression can modify amyloidogenic APP processing and Aβ secretion, and αSyn fibrils can perturb early-endosomal/endolysosomal function (Roberts et al. 2017; Zuo et al. 2026). These findings support biological plausibility but do not establish the same mechanism in SAA+ humans.

Mathematical models can provide a translational bridge between mechanisms tractable in cellular or preclinical systems and patterns measurable in living human cohorts. In AD, mechanistically informed models have tested whether experimentally supported protein-propagation hypotheses reproduce human tau-PET patterns, while quantitative systems pharmacology models integrate Aβ production, aggregation, transport, and clearance with human biomarker observations (Vogel et al. 2020; Schäfer et al. 2020; Lin et al. 2022; Ramakrishnan et al. 2023). Here the cellular literature defines plausible αSyn–APP/Aβ interactions, the cohort identifies the reproducible human phenotype, and the decomposition plus common-factor challenge asks which classes of perturbation are compatible with that phenotype and what measurements would discriminate among them.

The external associations make this soluble configuration biologically more informative than an algebraic decomposition. D42 was strongly related to subsequent fibrillar amyloid burden and was the more consistent dimension associated with subsequent tau burden and 0–2.5-year clinical worsening. T contributed little to clinical or tau-burden models, yet lower T independently predicted faster whole-brain loss in both cohorts and faster post-landmark amyloid-PET increase. T and D42 therefore are not interchangeable severity scores. D42 appears more closely aligned with the fibrillar/tau/clinical axis of AD, whereas the common shorter-Aβ dimension retains complementary information about structural degeneration and longitudinal fibrillar change.

This distinction is compatible with the emerging human literature in which SAA positivity has been associated with faster cognitive decline and SAA conversion with Aβ pathology (Tosun et al. 2024a, b), αSyn copathology with amplified amyloid-associated tau abnormalities and longitudinal tau accumulation (Franzmeier et al. 2025), and SAA positivity with cortical atrophy and microstructural decline (Esteller-Gauxax et al. 2026; Mak et al. 2026). Our direct SAA-to-clinical and SAA-to-tau estimates were imprecise, but the molecular coordinates address a different question: αSyn positivity may mark a context in which the amyloid system is configured differently before its consequences are captured by a single downstream outcome. The clearest direct SAA association was faster whole-brain loss; adding T_R and D42_R attenuated that coefficient by approximately 40%. This is not evidence of mediation, but indicates shared prognostic information between SAA status, the T-low/D42-high configuration, and structural degeneration.

The A/S description also argues against SAA positivity as merely a marker of more advanced AD: SAA+ participants occurred in both amyloid-PET-positive and amyloid-PET-negative groups. Within an Aβ-positive state, αSyn-associated alterations in trafficking or peptide disposition could modify how amyloid biology couples to tau or neurodegeneration; in Aβ-negative individuals, the same SAA signal may reflect another disease context. A particularly informative test would exploit longitudinal SAA conversion. Serial BACE1/sAPPβ and Aβ38/Aβ40/Aβ42 around conversion could determine whether the soluble system moves toward lower T and higher D42 as seeding becomes detectable. A shift without proportional proximal-marker change would move the candidate locus further downstream; parallel changes in APP C-terminal substrates or proximal readouts would implicate an earlier step. Expanded panels including Aβ37 and Aβ43 could help distinguish altered γ-secretase product generation or processivity from subsequent peptide-specific handling. No within-person shift would instead favor a stable predisposition or shared upstream factor.

The 2.5–5-year estimates add temporal caution but do not change the primary interpretation. Associations were heterogeneous and did not reproduce a coherent change in the dominant component across cohorts. Because these are follow-up intervals rather than biological stages, they should not be interpreted as an early-to-late mechanistic transition. Baseline soluble information may instead become diluted by accumulated downstream pathology, selective follow-up, and other time-varying processes, making denser longitudinal soluble sampling more informative than inferring intracellular sequence from distant outcomes.

Several limitations define the boundaries of these inferences. The proximal same-day cohort was small and followed an initial exploratory observation; the participant-disjoint replication lacked contemporaneous BACE1/sAPPβ and therefore cannot independently reproduce the complete proximal-to-soluble model. The common-factor benchmark represents one linear-Gaussian measurement structure rather than all possible dynamical systems. SAA is a binary marker of detectable seeding activity rather than total Lewy pathology; tau-PET availability was limited; subsequently measured PET or tau burden may include pathology already present at the molecular landmark; and D42 is a generated analytical coordinate rather than an assayed species. Only three participant-disjoint participants had later Aβ38/Aβ40/Aβ42 measurements, precluding direct soluble-trajectory analysis. These limitations prevent identification of a molecular reaction, but they define the measurements required to discriminate among the mechanisms suggested by the observed geometry.

In conclusion, CSF αSyn-SAA positivity identifies a reproducible T-low/D42-high soluble-Aβ configuration with distinct relationships to subsequent AD pathology and neurodegeneration. This pattern is difficult to reconcile with a simple uniform shift in proximal β-processing and instead points to altered post-β-cleavage APP/Aβ handling. D42 captures the component most closely aligned with fibrillar amyloid, tau burden, and clinical worsening, while the combined T-low/D42-high state is also associated with greater structural loss. Although the precise molecular step remains unresolved, the convergent findings support a post-β-cleavage locus for the αSyn-associated reorganization of Aβ dynamics.

## Supplementary Material

This is a list of supplementary files associated with this preprint. Click to download.

• OnlineResource1SupplementaryResultsJNT.pdf

## Figures and Tables

**Table 1:** Characteristics of the same-day proximal and participant-disjoint replication cohorts.

Characteristic	Stage 1 SAA− N = 59	Stage 1 SAA + N=13	Replication SAA− N = 429	Replication SAA + N=131
Age, years, mean (SD)	73.7 (8.3)	75.8 (9.3)	73.6 (7.4)	75.7 (7.5)
Female sex, n (%)	28 (47.5)	3 (23.1)	206 (48.0)	50 (38.2)
APOE ε4 carrier, n (%)	29 (49.2)	6 (46.2)	209 (48.7)	74 (56.5)
Education, years, mean (SD)	15.6 (2.9)	15.0 (3.2)	16.1 (2.6)	16.2 (2.8)
CN syndrome, n (%)	11 (18.6)	4 (30.8)	104 (24.3)	20 (15.3)
MCI syndrome, n (%)	31 (52.5)	6 (46.2)	216 (50.5)	52 (39.7)
AD dementia syndrome, n (%)	17 (28.8)	3 (23.1)	108 (25.2)	59 (45.0)

APOE ε4 carrier denotes ≥ 1 ε4 allele. Clinical syndrome labels are descriptive and were not treated as etiologic diagnoses. Replication diagnosis was available for 559/560 participants; SAA− diagnosis percentages use N = 428.

**Table 2:** Associations of αSyn-SAA positivity with proximal and soluble-Aβ measurements.

Outcome / contrast	Same-day Stage 1 β (95% CI)	P	Disjoint replication β (95% CI)	P
BACE1	−0.243 (−0.940, 0.454)	0.494	NA	NA
sAPPβ ∣ BACE1	−0.537 (−1.129, 0.055)	0.075	NA	NA
T / T_R	−0.549 (−1.005, −0.093)	0.018	−0.267 (−0.477, −0.057)	0.0128
D42 / D42_R	−0.223 (−0.782, 0.337)	0.436	0.206 (0.048, 0.365)	0.0108
Aβ38	−0.580 (−1.069, −0.092)	0.0198	−0.276 (−0.491, −0.061)	0.0117
Aβ40	−0.504 (−0.946, −0.062)	0.0255	−0.252 (−0.458, −0.047)	0.0162
Aβ42	−0.057 (−0.652, 0.538)	0.851	−0.313 (−0.493, −0.133)	0.00067
G38:40 / G38:40_R	−0.250 (−1.028, 0.528)	0.528	−0.084 (−0.295, 0.127)	0.434
D38_R	NA	NA	0.121 (−0.094, 0.336)	0.269
T-D42 direct contrast	−0.326; bootstrap interval − 1.159 to 0.517	-	−0.473 (−0.743, −0.204)	0.00058

Coefficients are adjusted differences for SAA + vs SAA− in outcome SD units unless otherwise indicated. Stage 1 soluble models condition on BACE1, sAPPβ, age, sex, APOE ε4 dosage, education, and syndrome; BACE1/sAPPβ models follow the frozen localization sequence. Replication models adjust for age, sex, APOE ε4 dosage, and education. The Stage-1 T-D42 interval is the prespecified participant-bootstrap interval; the replication contrast is fitted directly with HC3 covariance. NA, not available/not applicable.

**Table 3:** External associations of the common shorter-Aβ coordinate T and D42 in ADNI natural history.

Domain / outcome	Cohort / N	T effect	D42 effect
First subsequent amyloid PET	N560; N = 304	T_R: −2.19 (−6.45, 2.07) CL/SD; P = 0.314	D42_R: +34.90 (30.57, 39.23) CL/SD; P = 2.9×10^-56
CDR-SB worsening, 0-2.5 y	N380	T: +0.032 (−0.133, 0.197) points/y; P = 0.705	D42: +0.226 (0.085, 0.367) points/y; P = 0.0017
CDR-SB worsening, 0-2.5 y	N560	T_R: −0.018 (−0.140, 0.104) points/y; P = 0.773	D42_R: +0.241 (0.082, 0.401) points/y; P = 0.0030
ADAS-Cog13 worsening, 0-2.5 y	N560	T_R: +0.111 (−0.222, 0.445) points/y; P = 0.514	D42_R: +1.215 (0.621, 1.809) points/y; P = 6.1×10^-5
FAQ worsening, 0-2.5 y	N560	T_R: +0.172 (−0.109, 0.454) points/y; P = 0.231	D42_R: +0.604 (0.172, 1.035) points/y; P = 0.0061
MMSE worsening, 0-2.5 y	N560	T_R: −0.081 (−0.244, 0.081) points/y; P = 0.328	D42_R: +0.568 (0.258, 0.878) points/y; P = 0.00033
Whole-brain loss, 0-2.5 y	N380; N = 343	T: −0.144 (−0.237, −0.051) pp/y; P = 0.0025	D42: +0.180 (0.109, 0.250) pp/y; P = 5.2×10^-7
Whole-brain loss, 0-2.5 y	N560; N = 360	T_R: −0.077 (−0.142, −0.011) pp/y; P = 0.021	D42_R: +0.214 (0.107, 0.322) pp/y; P = 9.3×10^-5
First subsequent meta-temporal tau	N380; N = 28	T: −0.043 (−0.685, 0.599) tau SD; P = 0.895	D42: +0.904 (0.054, 1.754) tau SD; P = 0.037
First subsequent meta-temporal tau	N560; N = 96	T_R: +0.086 (−0.107, 0.279) tau SD; P = 0.380	D42_R: +0.525 (0.125, 0.924) tau SD; P = 0.010

Positive clinical coefficients denote faster worsening. Whole-brain loss is cumulative KN-BSI percentage-point loss per year; higher values indicate more atrophy. Tau effects are standardized outcome coefficients. Joint models condition on the other analytical components and demographic covariates. T denotes the common shorter-Aβ coordinate and is unrelated to AT(N) tau status. Complete estimates for the 0-2.5-year and 2.5-5-year follow-up intervals, formal contrasts between intervals, nonlinear tests, and sensitivity analyses are provided in Online Resource 1.

**Table 4:** Exploratory direct associations of SAA positivity with natural-history outcomes.

Outcome	N (SAA+)	Model A SAA + β (95% CI)	P	Model B + T_R/D42_R β (95% CI)	P
CDR-SB worsening	480 (116)	+ 0.178 (−0.162, 0.519)	0.305	+ 0.135 (−0.186, 0.456)	0.410
ADAS-Cog13 worsening	470 (112)	−0.075 (−1.179, 1.030)	0.894	−0.102 (−1.133, 0.929)	0.846
FAQ worsening	481 (116)	+ 0.710 (−0.159, 1.580)	0.109	+ 0.662 (−0.195, 1.519)	0.130
MMSE worsening	471 (112)	+ 0.281 (−0.279, 0.841)	0.325	+ 0.226 (−0.301, 0.752)	0.401
Whole-brain loss	360 (85)	+ 0.233 (0.041, 0.424)	0.0171	+ 0.140 (−0.047, 0.327)	0.142
Subsequent tau burden	96 (21)	−0.101 (−0.691, 0.489)	0.738	−0.089 (−0.684, 0.507)	0.771

Clinical and whole-brain-loss estimates are differences in worsening rate per year during the 0-2.5-year follow-up interval; subsequent tau burden is in tau-SD units. Model A uses the Stage-3 demographic/baseline covariate structure. Model B additionally conditions on T_R and D42_R. Coefficient attenuation is descriptive and is not a mediation estimate. Complete estimates for both longitudinal follow-up intervals, tau-PVC, and tau-progression bridge analyses are provided in Online Resource 1.

## Data Availability

The participant-level data used in this study were obtained from the Alzheimer’s Disease Neuroimaging Initiative (ADNI) and are available to approved investigators through the LONI Image and Data Archive, subject to the ADNI Data Use Agreement. Participant-level ADNI-derived data are not redistributed with this article.

## Code availability

Frozen analytical specifications, analysis scripts, preflight integrity checks, software requirements, and aggregate numerical outputs sufficient to reproduce the reported analyses after authorized retrieval of the corresponding ADNI source files are publicly available at Zenodo (Version 1.0.0; DOI: 10.5281/zenodo.21952005). The archived code does not contain participant-level ADNI data.
